# Overall survival in response to sorafenib versus radiotherapy in unresectable hepatocellular carcinoma with major portal vein tumor thrombosis: propensity score analysis

**DOI:** 10.1186/1471-230X-14-84

**Published:** 2014-05-03

**Authors:** Takahide Nakazawa, Hisashi Hidaka, Akitaka Shibuya, Yusuke Okuwaki, Yoshiaki Tanaka, Juichi Takada, Tsutomu Minamino, Masaaki Watanabe, Shigehiro Kokubu, Wasaburo Koizumi

**Affiliations:** 1 Department of Gastroenterology, Internal Medicine, Kitasato University School of Medicine, 2-1-1 Asamizodai, Minami-ku, Sagamihara, Kanagawa 252-0380, Japan; 2 Nakazawa Medical Clinic, Sagamihara, Japan; 3 Department of Gastroenterology, Juntendo University Nerima Hospital, Tokyo, Japan

**Keywords:** Hepatocellular carcinoma, Overall survival, Portal venous tumor thrombosis, Radiotherapy, Sorafenib

## Abstract

**Background:**

This study investigated the survival benefits of sorafenib vs. radiotherapy (RT) in patients with unresectable hepatocellular carcinoma (HCC) and portal vein tumor thrombosis (PVTT) in the main trunk or the first branch.

**Methods:**

Ninety-seven patients were retrospectively reviewed. Forty patients were enrolled by the Kanagawa Liver Study Group and received sorafenib, and 57 consecutive patients received RT in our hospital. Overall survival was compared between the two groups with PVTT by propensity score (PS) analysis. Factors associated with survival were evaluated by multivariate analysis.

**Results:**

The median treatment period with sorafenib was 45 days, while the median total radiation dose was 50 Gy. The Child-Pugh class and the level of invasion into hepatic large vessels were significantly more advanced in the RT group than in the sorafenib group. Median survival did not differ significantly between the sorafenib group (4.3 months) and the RT group (5.9 months; *P* = 0.115). After PS matching (n = 28 per group), better survival was noted in the RT group than in the sorafenib group (median survival, 10.9 vs. 4.8 months; *P* = 0.025). A Cox model showed that des-γ-carboxy prothrombin <1000 mAU/mL at enrollment and RT were significant independent predictors of survival in the PS model (*P* = 0.024, HR, 0.508; 95% CI, 0.282 to 0.915; and *P* = 0.007, HR, 0.434; 95% CI, 0.235 to 0.779; respectively).

**Conclusions:**

RT is a better first-line therapy than sorafenib in patients who have advanced unresectable HCC with PVTT.

## Background

Hepatocellular carcinoma (HCC) recurs frequently after curative treatment [1-4]. Advanced HCC sometimes causes macroscopic hepatic vascular invasion, including portal vein tumor thrombosis (PVTT) in the main portal trunk or the first branch and venous thrombosis in the hepatic vein trunk or inferior vena cava. These conditions can be life threatening, and the prognosis of patients with PVTT remains very poor, with a median survival of only approximately 3 months without treatment [5-8]. Therefore, identification of effective treatments that are not associated with significant adverse effects would be of benefit for this patient population. Transarterial chemoembolization (TACE) is one treatment for advanced HCC and is associated with an increased risk of ischemic necrosis of the liver and of treatment-related death in patients with PVTT. Therefore, this strategy is limited to a select group of patients with good hepatic function, patients with PVTT other than in the main or the first branch, and those with adequate collateral circulation around the occluded portal vein. Other treatment options include hepatic infusion chemotherapy mainly with 5-fluorouracil and cisplatin with or without interferon [9-11]. However, the efficacy of such treatments is limited, and this regimen can cause considerable stress for patients.

The use of molecular targeted therapy continues to increase. Sorafenib is an oral multikinase inhibitor with antiangiogenic and antiproliferative effects that significantly improves time-to-tumor progression and overall survival (OS) of patients with advanced HCC and is widely used to treat advanced HCC in which curative therapy is not indicated [12-14]. Sorafenib inhibits several tyrosine kinase receptors, including vascular endothelial growth factor (VEGF) receptor (R)-2, VEGFR-3, platelet-derived growth factor receptor β, FLT-3, and C-kit [15]. Although the use of sorafenib is limited to a select group of patients with good hepatic function, it can also be effective for patients with advanced HCC and a poor prognosis, including those with worse ECOG performance status, extrahepatic spread, vascular invasion, older age, and presence of macroscopic vascular invasion or extrahepatic spread [12]. However, care must be exercised due to the fact that sorafenib frequently causes various adverse events (AEs) such as hand-foot syndrome, gastrointestinal hemorrhage, and use-limiting anorexia [12-14].

Radiotherapy (RT) can produce survival benefits in patients with advanced HCC and macroscopic hepatic vascular invasion [7,16-18]. We previously reported that the use of three-dimensional conformal RT (3D-CRT) resulted in a good disease control rate and prolonged survival in these patients. Because of a high induction rate of stable disease (SD), both responders and nonresponders had improved outcomes when compared with patients who received supportive care alone [8,17]. Another advantage of 3D-CRT is that treatment can be administered on an outpatient basis without the difficulties associated with TACE or hepatic infusion chemotherapy, and RT did not produce grade 3 or higher liver, gastrointestinal, or hematological toxicity [8,17].

The goal of the present study was to compare the survival benefit of sorafenib versus RT in two retrospective cohorts of patients with advanced HCC and PVTT in the main trunk or the first branch. Propensity score analysis was used to reduce biases, and potential predictors of survival were analyzed using a Cox model.

## Methods

### Study population

Ninety-seven patients with macroscopic hepatic vascular invasion were retrospectively reviewed following approval by the institutional review board at Kitasato University East Hospital. Study protocols were conducted in accordance with the principles of the Declaration of Helsinki. All patients provided written, informed consent. HCC with macroscopic hepatic vascular invasion included patients with portal tumor invasion involving first-order branches and the main trunk of the portal vein, and venous thrombosis in the hepatic vein trunk or inferior vena cava. A diagnosis of tumor invasion and macroscopic hepatic vascular invasion was established in all patients by computed tomography (CT) on the basis of the following criteria: (i) a low-attenuation intraluminal filling defect with expanded macroscopic hepatic vascular invasion adjacent to the primary tumor during the portal phase, and (ii) an enhanced inner side of the filling defect during the arterial phase. Forty patients treated with sorafenib enrolled in the Kanagawa Liver Study Group (four institutes in Kanagawa Prefecture in Japan) and 57 consecutive HCC patients treated with RT in Kitasato University East Hospital (Sagamihara, Kanagawa, Japan) were examined. Overall survival (OS) and AEs were compared between the two groups of the entire cohort and in a PS-matched cohort. Factors potentially associated with OS were analyzed statistically in a PS-matched model. Treatment response was not compared, because the Response Evaluation Criteria in Solid Tumors (RECIST) criteria (version 1.1) and the modified RECIST criteria, which are commonly used for patients with HCC treated with sorafenib, were not adapted for use in patients with macroscopic hepatic vascular invasion [19,20]. The follow-up period was from initiation of treatment to the time of death. AEs were assessed according to the National Cancer Institute Common Terminology Criteria for Adverse Events, version 4.0.

### Sorafenib group

From July 2009 through November 2011, a total of 40 patients with advanced HCC with macroscopic hepatic vascular invasion and chronic liver disease of mainly Child-Pugh (C-P) class A received sorafenib at four institutes in Kanagawa Prefecture in Japan (Kitasato University East Hospital, Sagamihara; Yokohama City University Hospital, Yokohama; St. Marianna University Hospital, Kawasaki, and Kanagawa Cancer Center, Yokohama). Eligibility criteria for treatment with sorafenib were as follows: (i) unresectable advanced HCC without HCC rupture; (ii) no effect of TACE; (iii) no previous sorafenib therapy for the liver tumor; (iv) C-P class A or B (up to a score of 7 points) hepatic function; (v) an Eastern Cooperative Oncology Group (ECOG) performance status of 0–2 [21]; and (vi) the following laboratory findings: neutrophil count above 1500/μL, platelet count above 7.5 × 10^4^ mm^3^, and serum hemoglobin level above 8.5 g/dL. Patients initially received a standard dose of sorafenib, 400 mg twice daily (800 mg/day) or 200 mg twice daily (400 mg/day) for those with low body weight. The dose was reduced or treatment was temporarily suspended in patients who had drug-related grade 2–4 toxicities (until recovery to grade 1 or less) or at the discretion of the treating physician. The initial reduced dose of sorafenib was 400 mg/day. The dose was increased to the standard dose level in accordance with each patient’s tolerance. Treatment was continued until radiologic progression or recurrence of HCC, unacceptable toxicity associated with the study drug, or withdrawal of consent.

### RT group

From July 2001 through November 2011, 57 consecutive patients with advanced HCC and macroscopic hepatic vascular invasion initially received 3D-CRT at Kitasato University East Hospital, Sagamihara, Japan. Inclusion criteria for patients who received RT were as follows: (i) unresectable HCC with macroscopic hepatic vascular invasion; (ii) C-P class A or B hepatic function; (iii) an ECOG performance status of 0–2; (iv) no refractory ascites; and (v) no previous radiation therapy of the liver. The RT procedure was performed as described previously [8,17]. Briefly, macroscopic hepatic vascular invasion was mainly irradiated, regardless of the presence or absence of multinodular HCC. RT doses and treatment angles were determined with the use of a 3D-view technique to minimize critical organ injury. CT planning was used to determine radiation fields and the clinical target volume (CTV), which was defined as only the macroscopic hepatic vascular invasion. The main HCC was also irradiated together with hepatic vascular invasion if the tumor was directly involved. Other multiple nodules were not always included in the CTV. 3D-CRT was planned according to tentative guidelines to ensure that the normal liver volume irradiated with more than one half of the prescribed dose did not exceed 50% of the total liver volume. A daily radiation dose of 1.8 to 2.0 Gy was administered with a 6- or 10-MV X-rays using two- to four-port combinations. Five fractions were administered per week to deliver a total dose of around 50 Gy.

### Statistical analysis

The overall survival rates of patients who underwent sorafenib or RT were calculated from the date of diagnosis of macroscopic hepatic vascular invasion. The primary end point was all-cause mortality. The Chi-square or Fisher’s exact test was used to compare categorical variables, whereas Student’s *t-*test or the Mann–Whitney *U* test was used for continuous variables. The Kaplan-Meier method was used to obtain the cumulative survival rate. PS analysis was performed using multiple logistic regression to analyze patients treated with sorafenib or RT. Variables associated with treatment decisions were entered in the PS model. The PS model was then used to provide a one-to-one match between the sorafenib and RT groups by the nearest-neighbor matched method [22]. In each matched subgroup, survival curves were compared using the log-rank test. Variables that achieved significance (*P* < 0.05) or those that were close to significance (*P* < 0.15) by the log-rank test were subsequently included in the multivariate analysis using a forward stepwise Cox regression model for the analysis of factors associated with OS, with adjustments for confounding factors. A two-tailed *P* < 0.05 was considered significant. All statistical analyses were performed using the Statistical Package for Social Sciences (SPSS 17.0 for Windows, SPSS, Inc. Chicago, IL).

## Results

### Patient characteristics and crude OS in response to sorafenib versus RT

All patients (n = 97) underwent either sorafenib (n = 40) or RT (n = 57) treatment. In the sorafenib group, 28 patients initially received a dose 400 mg of sorafenib twice daily (800 mg/day), while 12 received a dose of 200 mg of sorafenib twice daily (400 mg/day) because of older age, low body mass index, or anorexia. The mean duration of treatment with sorafenib was 45 days (range, 7–400 days).

**Figure 1:**
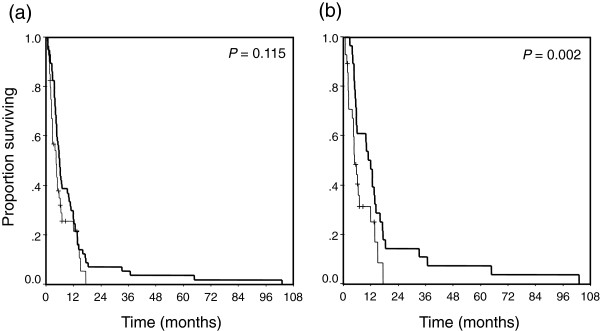
**Kaplan-Meier curves for OS of the RT (bold lines) and sorafenib (solid lines) groups. (a)**, all patients (n = 97) and **(b)**, PS-matched patients (n = 56). Although no significant difference between the two groups is observed in **(a)** (*P* = 0.115), OS in the PS-matched patients is significantly longer in the RT group (median 10.9 months) than in the sorafenib group (median 4.8 months), as shown in **(b)** (*P* = 0.002, log-rank test).

A total radiation dose of 30 to 56 Gy (median 50 Gy) was delivered, and a combination of PVTT and hepatic vein tumor thrombosis (HVTT) was observed in 10 patients in the RT group. The sorafenib group had significantly better hepatic function of C-P class A/B (sorafenib 36/4 and RT 34/23 patients, respectively, *P* = 0.001) and median platelet counts than the RT group (sorafenib 15.1 and RT 11.8 × 10^4^/mm^3^, respectively, *P* = 0.004). Tumor thrombosis in the main portal trunk was significantly more common in the RT group than in the sorafenib group (main/first branch: sorafenib 7/33 and RT 22/35 patients, respectively, *P* = 0.021). Otherwise, age, sex, the proportions of anti-hepatitis C virus-positive and of extrahepatic spread, and the median values of laboratory findings including α-fetoprotein and des-γ-carboxy prothrombin were not significantly different between the two groups. Thirty-three patients treated with sorafenib died (83%), while 57 treated with RT died during the observation period. Despite the fact that the RT study population had significant worsening of hepatic function and tumor progression in comparison with the sorafenib group, crude OS was not significantly different between the two groups [*P* = 0.115, 4.4 months (range, 0.7-17.5) in the sorafenib group, and 5.9 months (range, 0.6-103) in the RT group], as shown in Figure 1a.

### OS and factors related to OS in the PS-matched population

**Figure 2:**
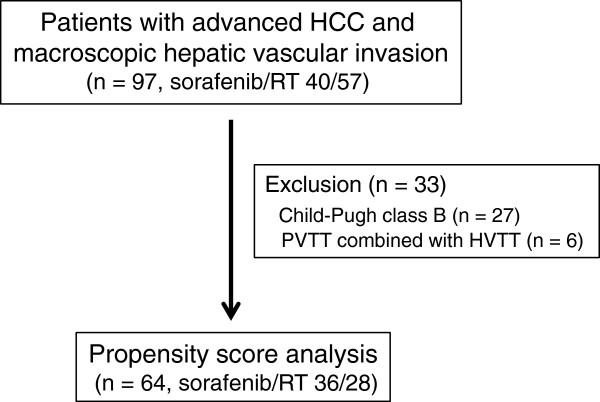
**Enrollment of patients.** HCC: hepatocellular carcinoma; RT: radiotherapy; PVTT: portal vein tumor thrombosis; HVTT: hepatic vein tumor thrombosis.

A total of 64 patients with C-P class A hepatic function and PVTT only. (sorafenib; n = 36, RT; n = 28) was extracted for PS analyses, as shown in Table 1 and Figure 2. Six of the 34 RT group patients with C-P class A were excluded because they had a combination of PVTT and HVTT. Significant differences between these two groups were observed in baseline levels of the tumor markers α-fetoprotein and des-γ-carboxy prothrombin (DCP). PS analysis with the one-to-one nearest-neighbor matching method was conducted to minimize selection bias and to adjust backgrounds. The two PS-matched groups (28 patients per group) were well balanced, as shown in Table 1. The PS-matched model was validated by the Hosmer and Lemeshow goodness-of-fit test (*P* = 0.091) and by the value of the area under the curve (0.719; 95% CI, 0.594-0.844). In the PS-matched cohort, the median OS was significantly shorter in the sorafenib group (4.8 months; range, 0.7-17.3) than in the RT group (10.9 months; range, 2.8-103; *P* = 0.002, log-rank test), as shown in Figure 1b. Cox regression analyses showed that DCP <1000 mAu/mL at pretreatment and RT were independent contributors to OS (*P* = 0.024; HR, 0.508; 95% CI, 0.282 to 0.915; *P* = 0.007; HR, 0.434; 95% CI, 0.235 to 0.779, respectively) (Table 2).

**Table 1:** Baseline characteristics of 64 patients with Child-Pugh class A and 56 patients matched by propensity score

	**Entire cohort**			**PS-matched cohort**		
	**Sorafenib**	**RT**	***P *** **value**	**Sorafenib**	**RT**	***P *** **value**
**Covariates**	**n = 36**	**n = 28**		**n = 28**	**n = 28**	
Age (years)	70 (62–78)	67 (61–71)	0.069	70 (61–78)	67 (61–70)	0.04
Sex (male/female)	31/5	19/9	0.127	23/5	19/9	0.355
HCV	19/17	17/11	0.615	16/12	17/11	1.0
*Main/first branch	7/29	9/19	0.262	7/24	9/19	0.205
Metastases (present/absent)	7/29	2/26	0.278	6/22	2/26	0.252
Previous Treatments (present/absent)	26/10	20/8	1.0	18/10	20/8	0.775
TACE/TAI	21	20		15	20	
RFA	3	0		2	0	
RT	2	0		1	0	
AFP (ng/dL)	1047 (44–5919)	43 (10–1096)	0.005	680 (37–3708)	43 (10–1096)	0.144
DCP (mAU/mL)	2915 (111–19706)	224 (33–2880)	0.013	2151 (58–10775)	224 (33–2880)	0.488

Data are presented as medians (range).

*Abbreviations*: *TACE* transarterial chemoembolization, *TAI* transarterial infusion chemotherapy, *RFA* radiofrequency ablation, *RT* radiotherapy, *HCV* hepatitis C virus, *AFP* α-fetoprotein, *DCP* des-γ-carboxy prothrombin, *AST* aspartate aminotransferase, *ALT* alanine aminotransferase, *PS* propensity score.

*Portal tumor invasion to the main trunk or first branch.

**Table 2:** Cox regression analysis of factors potentially related to overall survival

	**Log-rank test**		**Cox**		
**Covariates**	**n**	***P *** **value**	***P *** **value**	**HR**	**95% CI**
Age (≥70/<70 y)	33/23	0.355			
Sex (male/female)	42/14	0.424			
HCV (present/absent)	34/22	0.58			
*Main trunk (present/absent)	16/40	0.612			
Extrahepatic spread (present/absent)	5/51	0.278			
Previous treatments (present/absent)	38/18	0.546			
AFP ≥ 100 (ng/dL)	28/28	0.073			
DCP ≥ 1000 (mAU/mL)	27	0.066	0.024	1	
<1000	29			0.508	0.282, 0.915
Sorafenib	28	0.002	0.007	1	
RT	28			0.434	0.235, 0.779

*Abbreviations*: *HCV* hepatitis C virus, *AFP* α-fetoprotein, *DCP* des-γ-carboxy prothrombin, *RT* radiotherapy, *HR* hazard ratio, *CI* confidence interval.

*Portal invasion to the main trunk.

### Treatment tolerability

Treatment tolerability was analyzed by comparison of the AEs between the sorafenib and RT groups matched according to PS score. In the sorafenib group, 25 (90%) of 28 patients permanently discontinued sorafenib (due to AEs, n = 15; disease progression, n = 10). There was no radiographic or clinical evidence of pancreatitis, and there were no drug-related deaths. As shown in Table 3, AEs of grade 3 or more were observed in 19 patients, and almost all AEs were related to the liver (AST/ALT increase in six patients, anorexia/nausea in four patients, hepatic failure in one patient, and ascites in one patient). In the RT group, there was no grade 3 or higher gastrointestinal or hepatic toxicity, including anorexia/nausea, gastric ulcer, increase in AST/ALT, or hepatic failure. Grade 3 leukocytopenia was observed in only one patient. There were no long-term sequelae.

**Table 3:** Comparison of AEs between sorafenib and RT

	**Sorafenib**	**RT**
**Grade 3/4 toxicity**		
**Total (n)**	19	1
AST/ALT increased	6	0
Anorexia/nausea	4	0
HFSR	3	0
Hepatic Failure	1	0
Ascites	1	0
Hypertension	1	0
Proteinuria	1	0
Sepsis	1	0
Thrombocytopenia	1	0
Anemia	0	0
Leukocytopenia	0	1
Pancreatitis	0	0
**Discontinuation**, n (%)	15 (54)	1 (4)

*Abbreviations*: *AEs* adverse events, *AST* aspartate aminotransferase, *ALT* alanine aminotransferase, *RT* radiotherapy, *HFSR* hand-foot-skin reaction.

## Discussion

PS analysis demonstrated that RT was associated with better survival than sorafenib in patients with advanced unresectable HCC and PVTT. PVTT occurs in a substantial portion of HCC patients and is evident in up to approximately 40% of HCC patients at the time of death [7,9,23]. Sorafenib is an oral multikinase inhibitor that prolongs survival and the time to progression in patients with advanced HCC. This drug is also effective in patients with advanced HCC and poor prognosis, including those with worse ECOG performance status, extrahepatic spread, vascular invasion, older age, and the presence of macroscopic vascular invasion or extrahepatic spread [12]. In a recent PS analysis of sorafenib alone versus sorafenib combined with TACE for advanced HCC (in which 20-30% of the patient population had major trunk PVTT), neither regimen produced a significant benefit in OS [24]. We previously reported that RT produced favorable survival benefits without the hardships associated with conventional treatment for macroscopic hepatic vascular invasion [8,17].

The current study demonstrated that DCP <1000 mAu/mL at pretreatment and RT were independently related to OS, according to a Cox model in a PS analysis. The serum DCP level correlates with intrahepatic vascular invasion, and the DCP level might reflect expansion of macroscopic hepatic vascular invasion [25]. These findings suggest that the first goal of therapy for advanced HCC with major PVTT should consist of intensive treatment to recanalize the PVTT. RT is more effective than sorafenib, because major PVTT is intensively irradiated by RT. In fact, the overall objective response rate (complete response plus partial response) for PVTT by RT reached 45%, and the response rate was even better in patients with C-P class A [17]. In addition, 3D-CRT for PVTT can minimize liver-related AEs (Table 3). Almost all patients receiving sorafenib discontinued therapy (due to AEs or disease progression), while only one patient discontinued RT. Involvement of the main PVTT is associated with poor prognosis, possibly because of increased risk of tumor spread, elevated portal venous pressure causing variceal hemorrhage, and decreased portal flow resulting in ascites, jaundice, hepatic encephalopathy, and liver failure [7,9,23,26]. Sorafenib can compromise hepatic function by decreasing portal blood flow, as we previously demonstrated that sorafenib induced significant vasoconstriction of the portal venous area and significantly reduced portal venous flow, according to Doppler ultrasonography in patients with unresectable HCC [27]. Other investigators have used magnetic resonance imaging to show similar results [28]. Therefore, we believe that sorafenib should be administered only after recanalization of major PVTT by other treatments [9].

The optimal treatment regimen for patients with unresectable HCC and PVTT remains to be established. C-P class A hepatic function is likely related to the treatment response and survival, because it was previously identified as one of the factors contributing to OS in various treatments for HCC. Furthermore, we previously reported that C-P class A hepatic function was related to the response to RT [17]. Conversely, patients with C-P class B hepatic function tend to have a poor response to treatment, because treatments often further impair hepatic function.

Limitations of the current study include the small study population, as the number of patients with HCC and major PVTT is relatively small in the general population. Furthermore, this was a non-prospectively randomized study, and the evaluation of responsiveness to sorafenib may have been incomplete due to the involvement of different institutions. Therefore, OS and contributing factors were analyzed by PS analysis.

## Conclusions

First-line therapy for unresectable HCC with PVTT should consist of RT rather than sorafenib. Sorafenib should be introduced after recanalization of PVTT by other treatments, including RT. Multidisciplinary therapies based on individual hepatic function are expected to improve outcomes in the future.

## Abbreviations

RT: Radiotherapy; HCC: Hepatocellular carcinoma; PVTT: Portal vein tumor thrombosis; HVTT: Hepatic vein tumor thrombosis; PS: Propensity score; TACE: Transarterial chemoembolization; OS: Overall survival; VEGF: Vascular endothelial growth factor; AE: Adverse event; 3D-CRT: Three-dimensional conformal RT; RECIST: Response Evaluation Criteria in Solid Tumors; C-P: Child-Pugh; ECOG: Eastern Cooperative Oncology Group; CTV: Clinical target volume.

## Competing interests

The authors declare that they have no competing interests.

## Authors’ contributions

Conception and design: TN, HH, YO, YT, and JT; analysis: TN and YO; treatment and data collection: TN, HH, YO, YT, JT, and TM; drafting article: TN and AS; critical revision: MW, SK, and KW. All authors read and approved the final manuscript.

## Pre-publication history

The pre-publication history for this paper can be accessed here:

http://www.biomedcentral.com/1471-230X/14/84/prepub
